# *PMS2* amplification contributes brain metastasis from lung cancer

**DOI:** 10.1186/s12575-024-00238-1

**Published:** 2024-05-07

**Authors:** Jianing Chen, Congli Hu, Hainan Yang, Li Wang, Xiangling Chu, Xin Yu, Shiji Zhang, Xuefei Li, Chao Zhao, Lei Cheng, Weiping Hong, Da Liu, Lei Wen, Chunxia Su

**Affiliations:** 1 Department of Medical Oncology, Shanghai Pulmonary Hospital &, Thoracic Cancer Institute, Tongji University School of Medicine, Shanghai, China; 2 Department of Critical Care Medicine, Seventh People’s Hospital of Shanghai University of Traditional Chinese Medicine, Shanghai, China; 3 Department of Oncology, Guangdong Sanjiu Brain Hospital, Guangzhou, China; 4 Department of Neurosurgery, Guangdong Sanjiu Brain Hospital, Guangzhou, China; 5 Department of Radiation Oncology, Zhujiang Hospital, Southern Medical University, 253 Gongye Dadao, Guangdong, 510280 Guangzhou China; 6 Clinical Research Center, Shanghai Pulmonary Hospital, Shanghai, China

**Keywords:** Lung cancer, Brain metastasis, *PMS2*

## Abstract

**Background:**

Lung adenocarcinoma metastasizing to the brain results in a notable increase in patient mortality. The high incidence and its impact on survival presents a critical unmet need to develop an improved understanding of its mechanisms.

**Methods:**

To identify genes that drive brain metastasis of tumor cells, we collected cerebrospinal fluid samples and paired plasma samples from 114 lung adenocarcinoma patients with brain metastasis and performed 168 panel-targeted gene sequencing. We examined the biological behavior of *PMS2* (PMS1 Homolog 2)-amplified lung cancer cell lines through wound healing assays and migration assays. In vivo imaging techniques are used to detect fluorescent signals that colonize the mouse brain. RNA sequencing was used to compare differentially expressed genes between *PMS2* amplification and wild-type lung cancer cell lines.

**Results:**

We discovered that *PMS2* amplification was a plausible candidate driver of brain metastasis. Via in vivo and in vitro assays, we validated that *PMS2* amplified PC-9 and LLC lung cancer cells had strong migration and invasion capabilities.

The functional pathway of *PMS2* amplification of lung cancer cells is mainly enriched in thiamine, butanoate, glutathione metabolism.

**Conclusion:**

Tumor cells elevated expression of *PMS2* possess the capacity to augment the metastatic potential of lung cancer and establish colonies within the brain through metabolism pathways.

**Supplementary Information:**

The online version contains supplementary material available at 10.1186/s12575-024-00238-1.

## Introduction

Brain metastasis (BM) is a significant contributor to morbidity and mortality, carrying an unfavorable prognosis [1]. Common primary tumors giving rise to BM include breast cancer, lung cancer, and melanoma. Among these, lung cancer exhibits the highest incidence of BM ranging from 20 to 56%, far beyond other malignancies [2]. The median survival post-metastasis varies widely, spanning from 3 to 27 months [3]. It highlights an unmet need for enhanced comprehension and novel treatments.

Understanding the mechanisms underlying brain metastasis is imperative due to its prevalence and adverse impact on survival. It highlights an unmet need for enhanced comprehension and novel treatments. Intriguingly, some patients manifest brain metastasis even while their extracranial disease remains controlled [3]. This clinical disparity is partly explained by insufficient systemic therapeutic penetration of the blood–brain barrier, but unfortunately most of the mechanisms are under water. The exploration is hindered by the fact that patients with BM often encounter limitations in undergoing surgical resection of primary tumors, and excising intracranial metastatic tumors poses significant challenges.

Observations suggest the presence of additional potentially oncogenic alterations in brain metastases, contributing to the therapeutic response divergence seen in some cases. The evolutionary process, marked by mutations, may serve as precursors to tumor development and subsequent metastasis [4]. Observations suggest the presence of additional potentially oncogenic alterations in brain metastases, contributing to the therapeutic response divergence seen in some cases. This underscores the complexity of the metastatic process and emphasizes the necessity for in-depth exploration of underlying mechanisms to pave the way for improved therapeutic interventions.

Patients with BM often face limitations in undergoing surgical resection of primary tumors, and the excision of intracranial metastatic tumors poses significant challenges. Consequently, obtaining specimens for histopathological or biomarker studies becomes exceedingly difficult. Recognizing this constraint, circulating cell-free DNA (cfDNA) emerges as a promising avenue in cancer management.

Previous investigations have underscored the substantial value of cfDNA in plasma or other body fluids for cancer diagnosis and treatment [5, 6]. Notably, the analysis of driver mutations through ctDNA in plasma demonstrates clinical utility, particularly in epidermal growth factor receptor (EGFR)-mutated non-small-cell lung cancer (NSCLC). Given the inherent inaccessibility to intracranial tumor tissues, cfDNA derived from brain malignancies has garnered increased attention as a valuable resource in cancer management. Herein, cerebrospinal fluid (CSF), in direct contact with brain neoplasms, emerges as a more suitable and less invasive avenue for diagnosing brain tumors, circumventing the need for risky surgical procedures [7, 8]

Furthermore, CSF liquid biopsy proves informative, especially in patients with multiple brain metastases, as it can detect mutations not observed in the primary tumor [4]. The study conducted by Wu highlights the significant value of CSF as a source for liquid biopsy, demonstrating its efficacy in detecting actionable mutations in leptomeningeal metastasis. This emphasizes the potential of CSF analysis as a valuable tool in the diagnosis and management of brain metastases [9].

The *PMS2* gene, situated on chromosome 7p22 within a 16 kb region, comprises 15 exons and 862 codons [10]. Functionally, the *PMS2* protein forms a heterodimer with the MLH1 protein, constituting the MutLα complex, which collaborates with the MutSα complex in the primary repair of single-nucleotide mismatches [11]. As one of the DNA mismatch repair (MMR) genes, *PMS2* plays a crucial role in genomic stability. Notably, pathogenic heterozygous germline variants in *PMS2* have been associated with Lynch Syndrome (LS) [12–16],while mutations in MMR genes are frequently detected in various solid tumors [17–19].

Moreover, *PMS2* variants have been identified in primary brain tumors, with their presence reported in cerebrospinal fluid samples from glioma patients through exome sequencing [21–23]. The loss of *PMS2* is also correlated with poorer overall survival (OS) and progression-free survival (PFS) in lower grade astrocytomas [22]. Immunohistochemical analysis indicating the loss of *MLH1/PMS2* co-expression is associated with a lower tumor mutational burden (TMB) [24]. Additionally, the *PMS2* gene is implicated in the apoptotic pathway [25, 26].

The association of the *PMS2* gene with brain metastasis in non-small cell lung cancer has not been investigated to date. This comprehensive approach aims to shed light on the involvement of the *PMS2* gene in the complex landscape of brain metastasis in non-small cell lung cancer.

## Methods

### Patients enrollment and samples collection

One hundred fourteen patients with brain metastases were enrolled in our study, who had all been admitted to the Guangdong Sanjiu Brain Hospital between January 2019 and January 2022. Brain parenchymal metastases have been evaluated by MRI, while leptomeningeal metastases were diagnosed with MRI or Cerebrospinal fluid cytologic testing. Brain metastasis is identified by clinicians and MRI images are evaluated by two radiologists. This research was approved by the ethics committee of Guangdong Sanjiu Brain Hospital and conducted in accordance with the Declaration of Helsinki. Written informed consent was obtained from all patients. One hundred fourteen CSF samples acquisition was performed by the clinician through the lumbar puncture, 10 ml of CSF was collected during the procedure. Sixty-eight plasma samples were obtained within one week. 2 ml of CSF was used for the cytologic test, 8 ml of CSF and 8 ml of plasma were collected for NGS. 8 ml of CSF or plasma was collected in a standard ethylenediaminetetraacetic (EDTA) acid tube and incubated at room temperature for 2 h. The supernatant was transferred into a 15 ml centrifuge tube and was centrifuged at 16,000 g at 4 °C for 10 min. The supernatant was then transferred to a new tube and stored at − 80 °C for further analysis. All procedures were completed within two hours of sample collection.

### DNA extraction and Library construction, target sequencing

DNA extraction, library construction, and targeted sequencing with a commercial panel of the 168-gene panel (Burning Rock Biotech, Guangzhou, China) followed with routine processing described in previous studies [9, 27]. In brief, cfDNA was recovered from 5 ml of CSF or plasma using the QIAamp Circulating Nucleic Acid kit by Qiagen (Valencia, California, US). The Qubit 2.0 Fluorimeter (Carlsbad, California, US) was used to assess the Quantification of cfDNA. At least 50 ng of cfDNA is required to construct NGS library. Fragments between 200 and 400 bp from the sheared cfDNA were selected using Agencourt AMPure beads (Beckman Coulter, California, US), then hybridized with capture probes baits. PCR amplification was performed after hybridization selection with magnetic beads. Target capture was performed using a 168-gene targeted panel. A bioanalyzer high-sensitivity DNA kit was used to assess the quality and size of the fragments. Indexed samples were sequenced on the Nextseq 500 sequencer (Illumina, Inc., California, US) with pair-end reads.

### Cell culture

Seven human lung cancer cell lines, namely H1299, A549, H1650, HCC827, H3255, H1975, and PC-9, along with the human bronchial epithelial cell line BEAS-2B and the Lewis lung carcinoma cell line, were obtained from the American Type Culture Collection (ATCC). These cell lines were cultured in either Roswell Park Memorial Institute (RPMI) 1640 medium (Biosharp, China) or Dulbecco's Modified Eagle Medium (DMEM) (Biosharp, China), both supplemented with 10% fetal bovine serum (FBS, Pricella, China) and 100 U/ml of a streptomycin/penicillin combination (Gibco; Thermo Fisher Scientific, Inc.). The cultures were maintained in a humidified atmosphere containing 5% CO2 at 37 °C.

### Lentivirus and transfections

A lentivirus overexpressing PMS2 was generated through lentiviral transduction utilizing the pcSLenti-EF1-EGFP-P2A-Puro-CMV-MCS-3xFLAG-WPRE vector provided by OBIO Technology (Shanghai) Corp., LTD. To establish a control, a lentivirus lacking the *PMS2* construct (designated LV-control) was created using an empty vector, pcSLenti-EF1-EGFP-P2A-Puro-CMV-PMS2-3xFLAG-WPRE. Additionally, a lentivirus overexpressing luciferase was generated by lentiviral transduction employing the pSLenti-EF1-Luc2-P2A-BSR-CMV-MCS-WPRE vector. The transfection process followed the manufacturer's instructions, with polybrene serving as the transfection agent. This standardized procedure ensures the efficient introduction of genetic material into the target cells, facilitating the overexpression of *PMS2* or luciferase as intended.

### Wound healing assay

Transfected cells were seeded in 6-well plates at a concentration of 2.5 × 10^5 cells/mL. Subsequently, cell monolayers were mechanically wounded using a sterile pipette tip (10 μL) to create a gap. The cells were then washed with 1X phosphate-buffered saline (PBS), and the remaining cells were cultured continuously in serum-free medium for 24 h. Representative fields were photographed, and the migrated cells were quantified by counting. This standardized protocol ensures consistent and reproducible assessment of cell migration following the specified experimental conditions.

### Transwell assay

Invasion assessment was conducted using 24-well Transwell inserts (8 μm aperture, BD Biosciences) pre-coated with Matrigel matrix (Corning, NY). Approximately 2 × 10^4 cells, suspended in serum-free medium, were seeded into the upper chambers, while the lower chambers were filled with DMEM containing 10% FBS. Cells were maintained at 37 °C throughout the experiment. After 24 h, non-invading cells on the upper side of the inserts were gently wiped off using a cotton swab. Subsequently, cells that had invaded through the Matrigel and reached the lower chambers were treated with 0.1% crystal violet and counted. This method provides a standardized approach for assessing cell invasion capabilities, ensuring reliable and reproducible results.

### RNA extraction and real-time quantitative PCR

Total RNA extraction was conducted using TRIzol reagent. Subsequently, cDNA was synthesized utilizing the PrimeScript™ RT Master Mix cDNA Synthesis Kit (RR360A, TaKaRa, China), following the manufacturer’s protocol. Real-time PCR was performed with TB Green qPCR Master Mix (RR820A, Takara, China) on the QuantStudio 6 Flex Real-Time PCR System (Thermo Fisher Scientific, Waltham, MA, USA). For normalization, relative mRNA levels were assessed based on the expression of *GADPH*. The expression values were analyzed using the 2 − ΔΔCt method, a widely accepted relative quantitative approach in real-time PCR studies. The primer sequences used were as follows: *PMS2* forward: 5′- CTGGATGCTGGTCCACTAA-3′, reverse: 5′- TGTGTGATGTTTCAGAGTTAAGCC-3′; *GAPDH* forward: 5′- ACAACTTTGGTATCGTGGAAGG -3′, reverse: 5′- GCCATCACGCCACAGTTTC -3′.

### Western blot analysis

Cells were lysed by cold RIPA Lysis Buffer (EpiZyme, China) supplemented with protease inhibitor on ice. Samples were boiled with SDS/PAGE sample buffer for 10 min and then were separated on SDS-PAGE. Post transferring, PVDF membranes (Millipore) were blocked by 5% non-fat milk TBST buffer, followed by incubation with primary antibodies at 4 °C overnight. After incubation with horseradish-peroxidase-coupled secondary antibodies at room temperature for 2 h, immunoreactive bands were visualized using ECL kit (EpiZyme, China). Anti-*PMS2* antibody (A4577) and anti-*GAPDH* (AC001) were purchased from ABclonal Technology Co., Ltd.

### In vivo mouse studies

We purchased 22 five-week-old female nude mice and mice were randomly assigned to experimental group and control group for all the experiments. After one week’s adaption, mice were anesthetized and injected with lung cancer cells (PC-9 control and *PMS2* amplificated PC-9 cells: 10^5/mouse) into the left cardiac ventricle as reported previously [28].

### Bioluminescence imaging (BLI)

For in vivo imaging, mice were anesthetized using 3% isoflurane in 100% oxygen. Subsequently, they were injected with 300 mg/kg of the substrate D-luciferin. The mice were then placed in the IVIS Spectrum instrument (Tanon 6600) to monitor the systemic dissemination of tumor cells. Images were captured at 5-min intervals until the photon counts reached their peak.

### DNA sequencing and analysis

DNA Sequencing data were mapped to the reference human genome (hg19) using Burrows-Wheeler Aligner version 0.7.10 [29]. Local alignment optimization, variant calling, and annotation were performed using GATK 3.2, MuTect, and VarScan. Full description was provided in a previous study [9].

### RNA sequencing and differentially expressed genes analysis

The libraries were sequenced on the llumina Novaseq 6000 platform and 150 bp paired-end reads were generated. Raw reads of fastq format were firstly processed using fastp and the low quality reads were removed to obtain the clean reads. Then clean reads for each sample were retained for subsequent analyses. The clean reads were mapped to the reference genome using HISAT2. FPKM of each gene was calculated and the read counts of each gene were obtained by HTSeq-count. PCA analysis were performed to evaluate the biological duplication of samples. Differential expression analysis was performed using the DESeq2. Q value < 0.05 and foldchange > 2 or foldchange < 0.5 was set as the threshold for significantly differential expression gene (DEGs). Based on the hypergeometric distribution, KEGG pathway enrichment analysis of DEGs was performed to screen the significant enriched term, respectively.

### Statistical analysis

Fisher’s exact test was used to compare the proportions between the two groups. Kaplan–Meier analysis was used to analyse the OS of each group and the log-rank test was performed to test survival differences between two groups. Besides, we performed all statistical analyses and plotting using the software package R version 4.2.3. The two-sided *p* < 0.05 was considered significant.

## Results

### Patients’ characteristics

We included 114 patients with brain metastases from non-small cell lung cancer, including 112 adenocarcinomas, 1 squamous cell carcinoma, and 1 adenosquamous cell carcinoma. Of the 114 patients included in the study, 28 had brain parenchymal metastases (BPM), including 18 who also had leptomeningeal metastases (LM). The rest of the patients presented with a combination of BPM and LM. The clinical characteristic of these patients was summarized in Supplemental Table 1.

### *PMS2* amplification identified in CSF samples

**Fig. 1:**
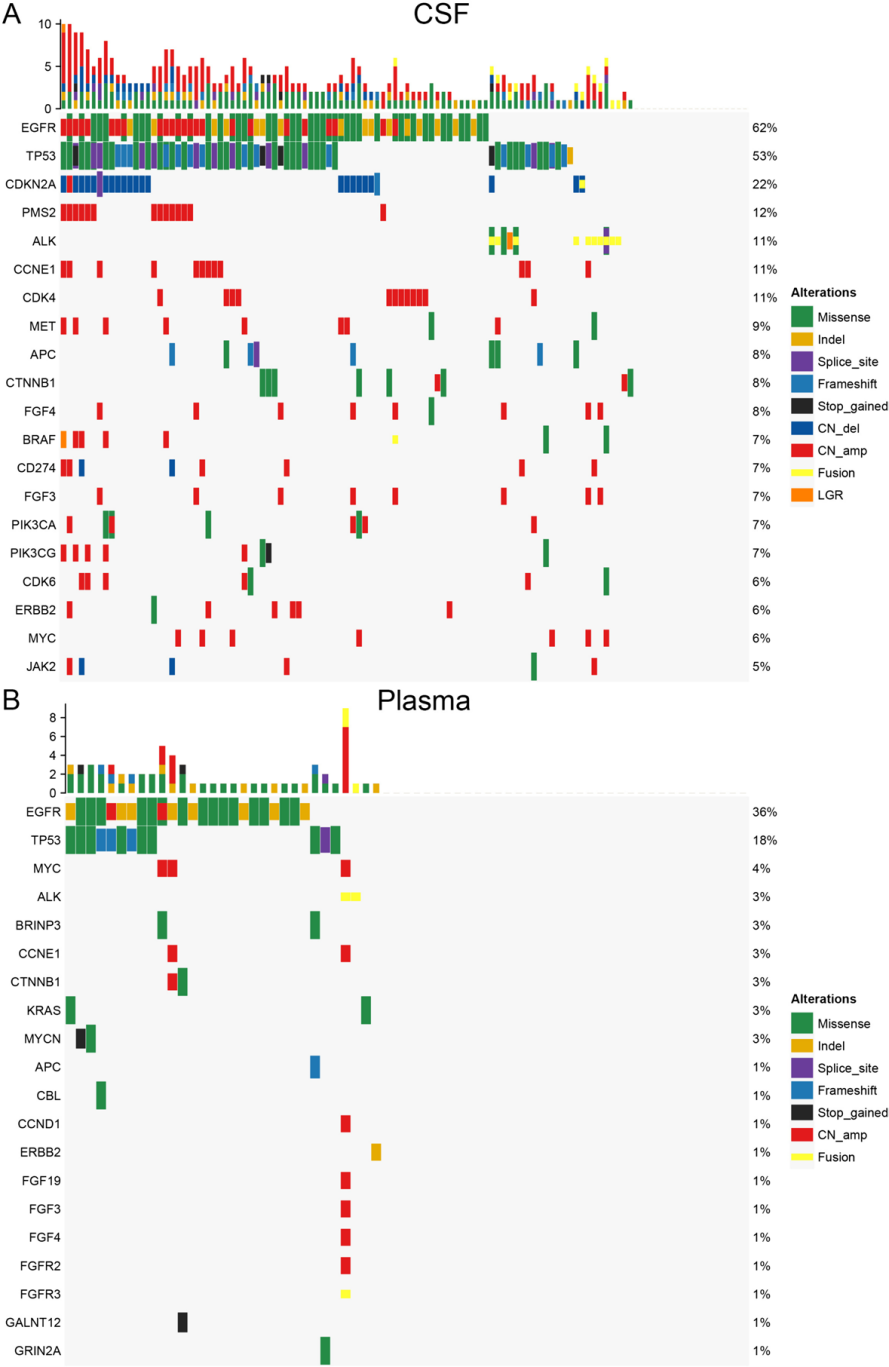
Genomic atlas of samples from patients with brain metastases. **A** genomic alternation detected in the cerebrospinal fluid. **B** genomic alternation detected in the plasma

*EGFR, TP53, CDKN2A, PMS2* and *CDK4* are the top five most common mutations in cerebrospinal fluid samples from patients with brain metastases (Fig. 1A), while *EGFR, TP53, MYC, ALK* and *BRINP3* are most frequently detected in plasma samples (Fig. 1B). Comparisons of the mutational landscape of CSF samples with that of plasma revealed that *PMS2* gene mutations in tumor cells are identified in a copy-number amplified form in the cerebrospinal fluid, no mutations in the *PMS2* gene were detected in the plasma. Besides, in previous article, similar result was reported that *PMS2* is a high frequently mutated genes in CSF compared with primary lung tumor tissues [30]. We analyzed the genomic characteristics of 337 primary early-stage lung adenocarcinomas from patients in the TCGA M0 Stage LUAD cohort, revealing an absence of *PMS2* alterations (Supplemental Fig. 1). These results suggest that *PMS2* amplification is specifically detected in the cerebrospinal fluid of patients with brain metastases. We then divided patients in our cohort into *PMS2* amplified and unamplified groups, the two group had a similar long-term follow-up overall survival (OS) (*P* = 0.57) (Supplement Fig. 2).

**Fig. 2:**
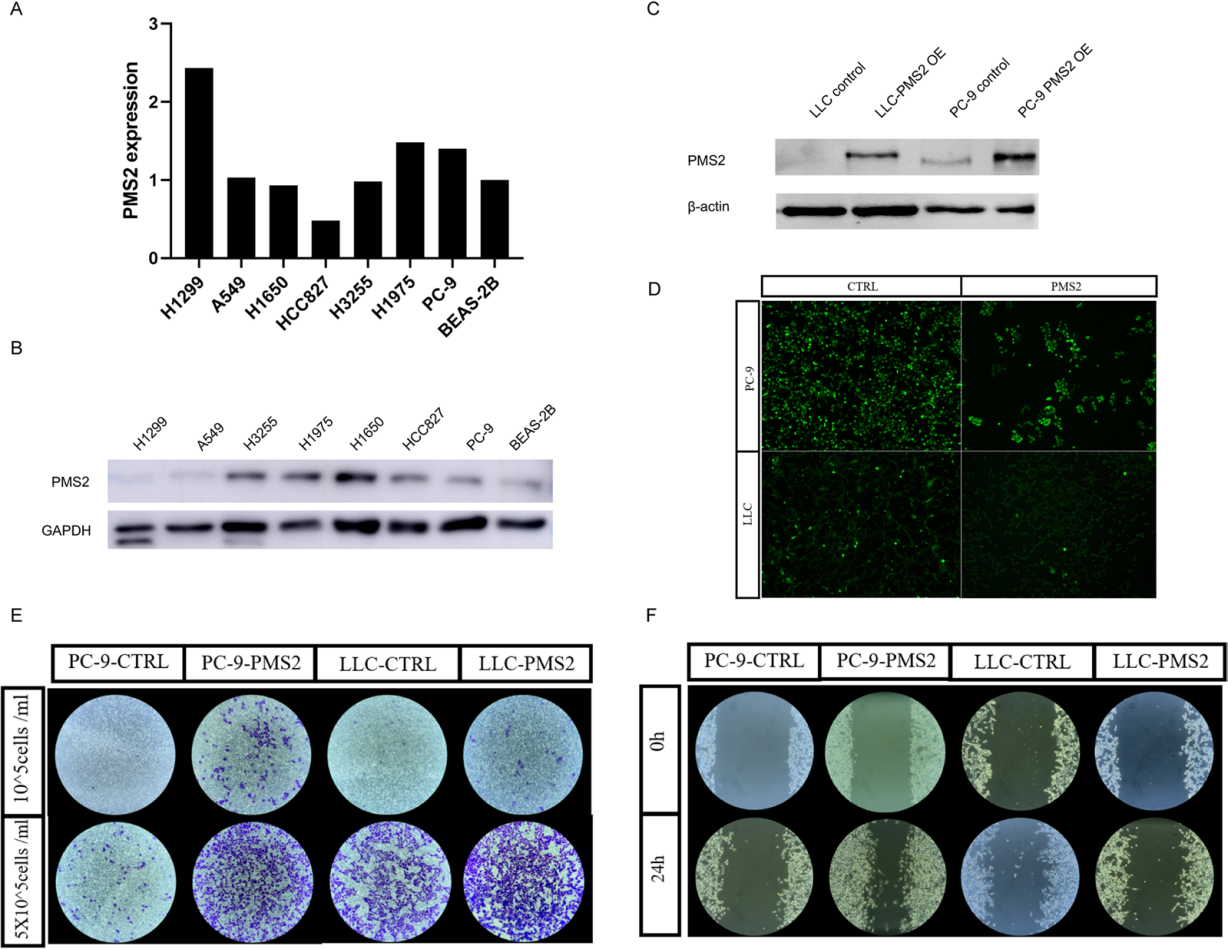
The expression level and cell migration and invasion abilities of *PMS2* in cell lines. **A** The relative transcriptional expression level of *PMS2* in in the human bronchial epithelial cell line BEAS-2B and seven lung cancer cell lines. **B** The protein expression levels of *PMS2* in in the human bronchial epithelial cell line BEAS-2B and seven lung cancer cell lines. **C** *PMS2* protein expression levels in transgenic cell lines. **D** Fluorescence photograph of lentivirus-infected lung cancer cell lines. **E** Transwell invasion assay and **F** Wound healing assay

### *PMS2* promotes the migration and invasion of lung cancer cells

Migration is a vital step for tumor cells to move from the primary lesion to distant organs. In order to investigate the effect of *PMS2* gene on the migration ability of lung cancer cells, we screened lung cancer cell lines by PCR (Fig. 2A) and western blotting (Fig. 2B, Supplement Fig. 3A) and we found PC9 lung cancer cell lines with consistently low *PMS2* expressed in at the transcriptional and protein levels. We further overexpressed *PMS2* in PC9 cell lines by lentiviral infection system. At the same time, we introduced the *PMS2* gene into a murine lung cancer LLC cell line (Fig. 2C,D, Supplement Fig. 3B). Through invasion experiments, we found that PMS2- amplificated tumor cells had a stronger invasion ability than control cells (Fig. 2E). Then we performed the wound healing assay, compared to the control cancer cell lines, *PMS2*-amplificated lung cancer cells had a stronger ability to migrate (Fig. 2F).

### *PMS2* overexpression stimulates tumor cells colonization in mice brains

**Fig. 3:**
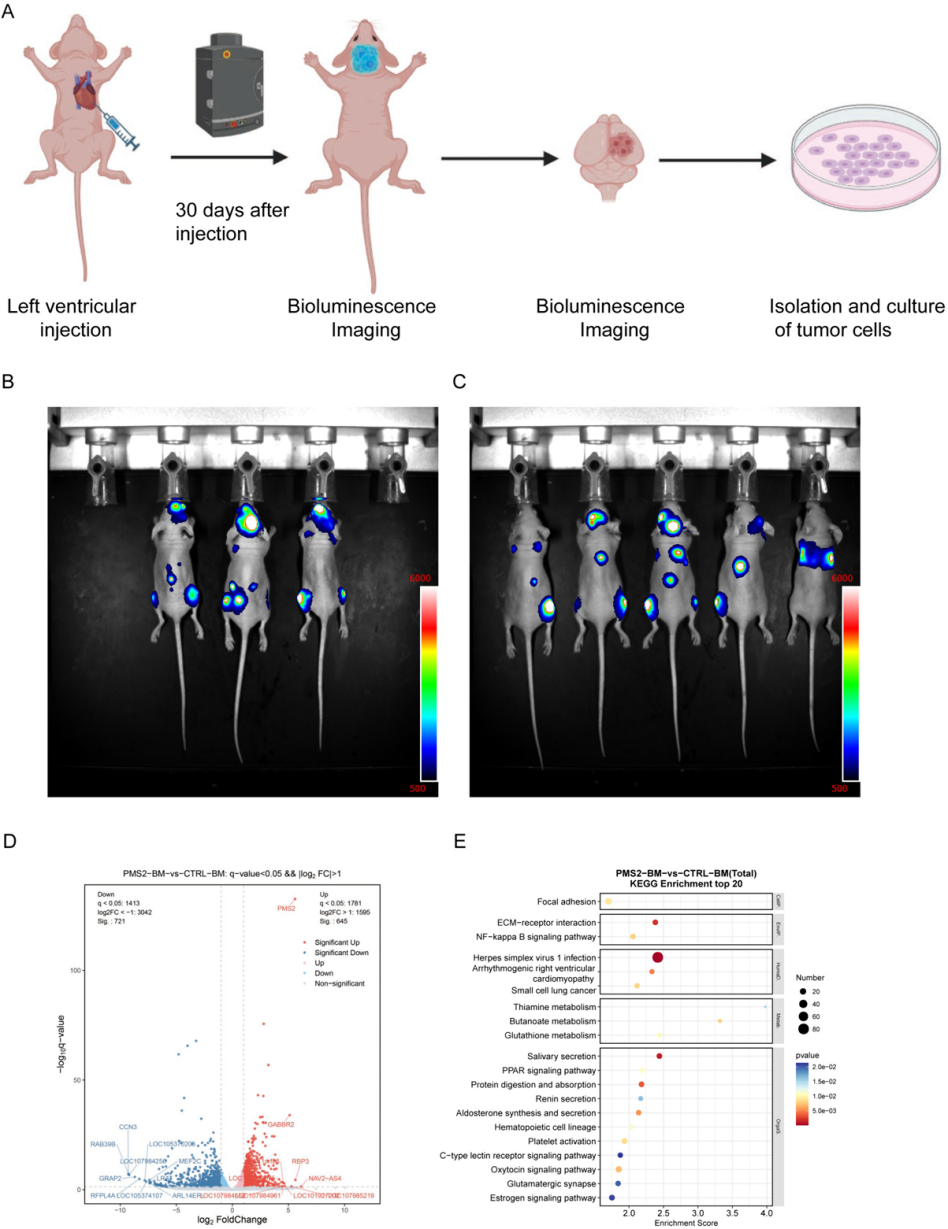
The Mechanism of *PMS2* Promoting Brain Metastasis. **A** Xenograft Model Flow Diagram. **B** Bioluminescence imaging of *PMS2* overexpressed mice. **C** Bioluminescence imaging of control mice. **D** Volcano plot of differentially expressed genes in tumor cells in the *PMS2* overexpression group versus the control group. **E** Functional enrichment dot plot of differentially expressed genes

To investigate the role of *PMS2* in brain metastasis, we tagged tumor cells with a dual luciferase labeling system to apply for tracking of tumor cells, and injected luciferase-labeled lung cancer cells into left ventricle of nude mice (Fig. 3A). We found that the mortality rate was higher in the *PMS2* amplified group (Supplement Fig. 4). By quantifying the fluorescence signal by in vivo Bioluminescence imaging (BLI) and comparing the signal intensity between the two groups (Fig. 3B-C), we found that the brain signals were stronger in the experimental group, but there was no statistical difference (Supplement Fig. 5). This may be due to the higher mortality rate of tumor cells after colonization of tumor cells overexpressing PMS2 in the mouse brain. Next, we sacrificed the mice with fluorescent signals in the brain in the two groups, separated the whole brains of mice, and extracted tumor cells from the brains for further culture. RNA sequencing was performed on tumor cells in *PMS2* amplification and control groups. We confirmed that expression level of *PMS2* was elevated in *PMS2* amplificated tumor cells (Fig. 3D). Functional enrichment analysis showed that the main pathways of the differentially differentiated genes between the two groups were concentrated in the thiamine, butanoate, glutathione metabolism (Fig. 3E). Furthermore, when conducting RNA sequencing on lung cancer cells before and after intracardiac injection without the transfer of the PMS2 gene, it was found that there were no enriched pathways related to metabolism (Supplement Fig. 6).

## Discussion

The process and molecular mechanism underlying the metastasis and colonization of lung cancer tumor cells in the brain remain incompletely understood. Genetic divergence between brain metastases and primary tumors has been reported [31]. The formation of brain tumors results from a series of genetic changes, playing a pivotal role in tumor evolution [32]. Genomic analysis of brain metastases offers an opportunity to identify clinically informative alterations not detected in primary tumors sampled during routine clinical procedures [4]. Therefore, discovering new genes is crucial for a better understanding of tumor development and progression.

*HBEGF*, a ligand for *EGFR*, has been specifically identified in brain metastasis and demonstrated to enhance invasion in breast cancer cells. Another brain metastasis-specific gene, *ST6GALNAC5*, mediates the interaction of cancer cells with brain endothelial cells [33]. In this context, our study identified *PMS2* as a potential driver gene for brain metastasis by sequencing cerebrospinal fluid and plasma and comparing genes with high-frequency mutations.

Approximately 4–5% of NSCLCs exhibit alterations in the genes constituting the mismatch repair (MMR) system [34]. The MMR system is crucial for DNA replication fidelity, involving seven known genes (*MLH1, MLH3, MSH2, MSH6, PMS1, PMS2,* and *EPCAM*) that recognize and repair single base–base mismatches or insertion–deletion loops [35, 36]. Mutations in these genes elevate susceptibility to various cancers [37, 38]. Loss of function in any MMR gene results in hypermutation and high microsatellite instability (MSI-H), causing errors in DNA replication and recombination [35, 39]. Dysregulation of mismatch repair gene expression, both loss and overexpression, can be detrimental to genomic stability, with loss-of-function mutations correlating with high TMB [40–42]. Overexpression of the *PMS2* gene disrupts MMR function, establishing an additional carcinogenic mechanism leading to genetic instability and resistance to cytotoxic cancer therapy [43]. Our gain-of-function studies reveal that *PMS2* overexpression increases the migration and invasion capabilities of cancer cells, crucial for the initiation of metastasis. Organ colonization emerged as a main rate-limiting step in the metastatic cascade, through BLI, our cell-derived xenograft mouse models validated that *PMS2* overexpression increased the brain metastasis incidence, compared to the control group. These results demonstrate that *PMS2* amplification is critical to brain metastasis formation by lung cancer cells.

The development of brain metastasis hinges on intricate interactions between cancer cells and the tumor microenvironment. The blood–brain barrier (BBB)/blood tumor barrier (BTB) imposes restrictions on nutrient access from circulation [44], resulting in a microenvironment characterized by hypoxia and depletion of essential metabolites, growth factors, and proteins [45]. Consequently, metastasized tumor cells undergo genetic and epigenetic changes to better adapt to this challenging environment [46–50]. Breast cancer cells that metastasize to the brain demonstrate a unique ability to utilize gluconeogenesis and oxidize branched-chain amino acids for growth, independent of glucose availability [51]. Likewise, limited microenvironmental serine and glycine results in selection of brain metastatic cells with increased dependency on de novo serine synthesis. Additionally, in response to limited microenvironmental serine and glycine, brain metastatic cells exhibit an increased dependency on de novo serine synthesis [52]. Brain-tropic breast cancer lines undergo reprogrammed lipid metabolism, including alterations in lipid transport, synthesis, and beta-oxidation [53–55]. Notably, lactate secretion by highly metastatic cells serves to limit innate immune surveillance and promotes significant metastases [56]. Overall, accumulating evidence underscores the crucial role of dynamic interplay between brain metastatic cancer cells and the surrounding immune microenvironment in the process of brain metastatic colonization [57–59].

It is reasonable to hypothesize that *PMS2* amplification occurs in tumor cells after entering the brain tissue through blood circulation, enabling better adaptation to the brain microenvironment. Transcriptome sequencing of *PMS2*-amplificated tumor cells colonized in the brain, compared to tumor cells in the control group, reveals enrichment in metabolic pathways, particularly involving glutamine, thiamine, and methyl butyrate. These findings shed light on the functional implications of PMS2 amplification in tumor cells during brain metastatic colonization, providing insights into potential metabolic adaptations that contribute to the successful establishment of brain metastases.

Our data provide new insights into complex interactions between brain metastatic cancer cells and DNA mismatch genes during metastatic colonization in the brain. However, our study still had four shortcomings. First, samples of patients with brain metastases were collected retrospectively from only one hospital. Second, Lack of brain tumor tissue samples to verify the reliability of our cerebrospinal fluid results. Third, the number of gene panel tests is small and may miss some genes related to brain metastasis. Last, current models of brain metastasis are largely based on hematogenous cancer cell dissemination upon intra-arterial or cardiac injection, thereby bypassing spontaneous dissemination from primary site [60, 61].

## Conclusion

In summary, this work demonstrated that somatic alterations of *PMS2* contribute to brain metastases and provides compelling evidence that *PMS2* upregulation is required for the tumor metastasizing to brain. We also suggested the potential of *PMS2* targeting for therapeutic intervention for life-threatening brain metastases.

### Supplementary Information

**Additional file 1:** **Supplemental Table 1.** Clinical characteristics of patients with brain metastases.

**Additional file 2:** **Supplemental Figure 1.** Waterfall diagram of gene mutations in tumor samples of M0 patients in TCGA database.

**Additional file 3:** **Supplemental Figure 2.** Overall survival plot of *PMS2* amplification positive and negative patients.

**Additional file 4:** **Supplemental Figure 3.** The protein level of cell lines. (A) the ratio of PMS2 /GAPDH density in eight cell lines. (B) the ratio of PMS2 /β-actin density in transgenic lung cancer cell lines.

**Additional file 5:** **Supplemental Figure 4.** Overall survival of mice after intracardiac injection. 

**Additional file 6:** **Supplemental Figure 5.** Histogram of fluorescence signal intensity in mouse brain. 

**Additional file 7:** **Supplemental Figure 6.** Functional enrichment plot of PC9 cells before and after intracardiac injection. 

## Data Availability

No datasets were generated or analysed during the current study.

## Abbreviations

BM: Brain metastasis
cfDNA: circulating cell-free DNA
NSCLC: Non-small cell lung cancer
EGFR: Epidermal growth factor receptor
CNS: Central nervous system
CSF: Cerebrospinal fluid
MMR: Mismatch repair
LS: Lynch Syndrome
OS: Overall survival
PFS: Progression-free survival
TMB: Tumor mutational burden
EDTA: Ethylenediaminetetraacetic
BLI: Bioluminescence imaging
MSI-H: High microsatellite instability
BBB: Blood–brain barrier
BTB: Blood tumor barrier
