# Supplementary material for: PMS2 amplification contributes brain metastasis from lung cancer
Source: Biol Proced Online. 2024 May 7;26:12. doi: 10.1186/s12575-024-00238-1 (PMC11075212; doi:10.1186/s12575-024-00238-1)
Supplement: Supplementary file 1 — Additional file 1: Supplemental Table 1. Clinical characteristics of patients with brain metastases. [file 12575_2024_238_MOESM1_ESM.docx]

|  | N=114 |
| --- | --- |
| **Gender** |  |
| Male | 62 |
| Female | 52 |
| **Age** |  |
| <65 | 93 |
| >=65 | 21 |
| **KPS score median(IQR)** | 70(50-80) |
| **Pathology** |  |
| adenocarcinoma | 112 |
| Squamous cell carcinoma | 1 |
| Adenosquamous carcinoma | 1 |
| Stage at initial diagnosis |  |
| I-III | 28 |
| IV | 86 |
| Site of metastases |  |
| Brain parenchymal metastases (BPM) | 28 |
| Leptomeningeal Metastases (LM) | 18 |
| BPM and LM | 68 |
| Evolution of metastasis |  |
| Synchronous | 54 |
| metachronous | 60 |
