# Supplementary figures and images for: PMS2 amplification contributes brain metastasis from lung cancer
Source: Biol Proced Online. 2024 May 7;26:12. doi: 10.1186/s12575-024-00238-1 (PMC11075212; doi:10.1186/s12575-024-00238-1)

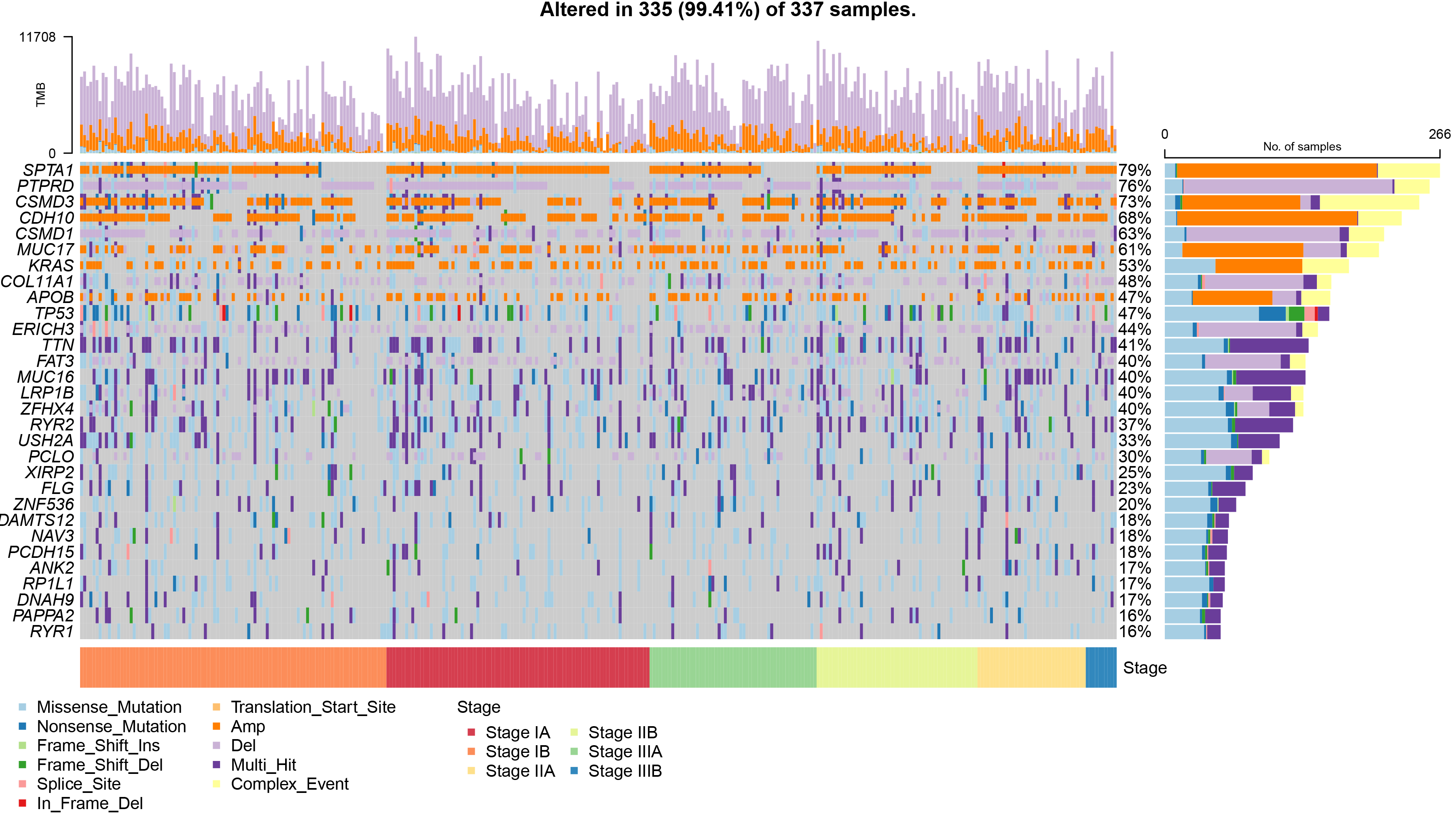

Supplement: Supplementary file 2 — Additional file 2: Supplemental Figure 1. Waterfall diagram of gene mutations in tumor samples of M0 patients in TCGA database. [file 12575_2024_238_MOESM2_ESM.tif]

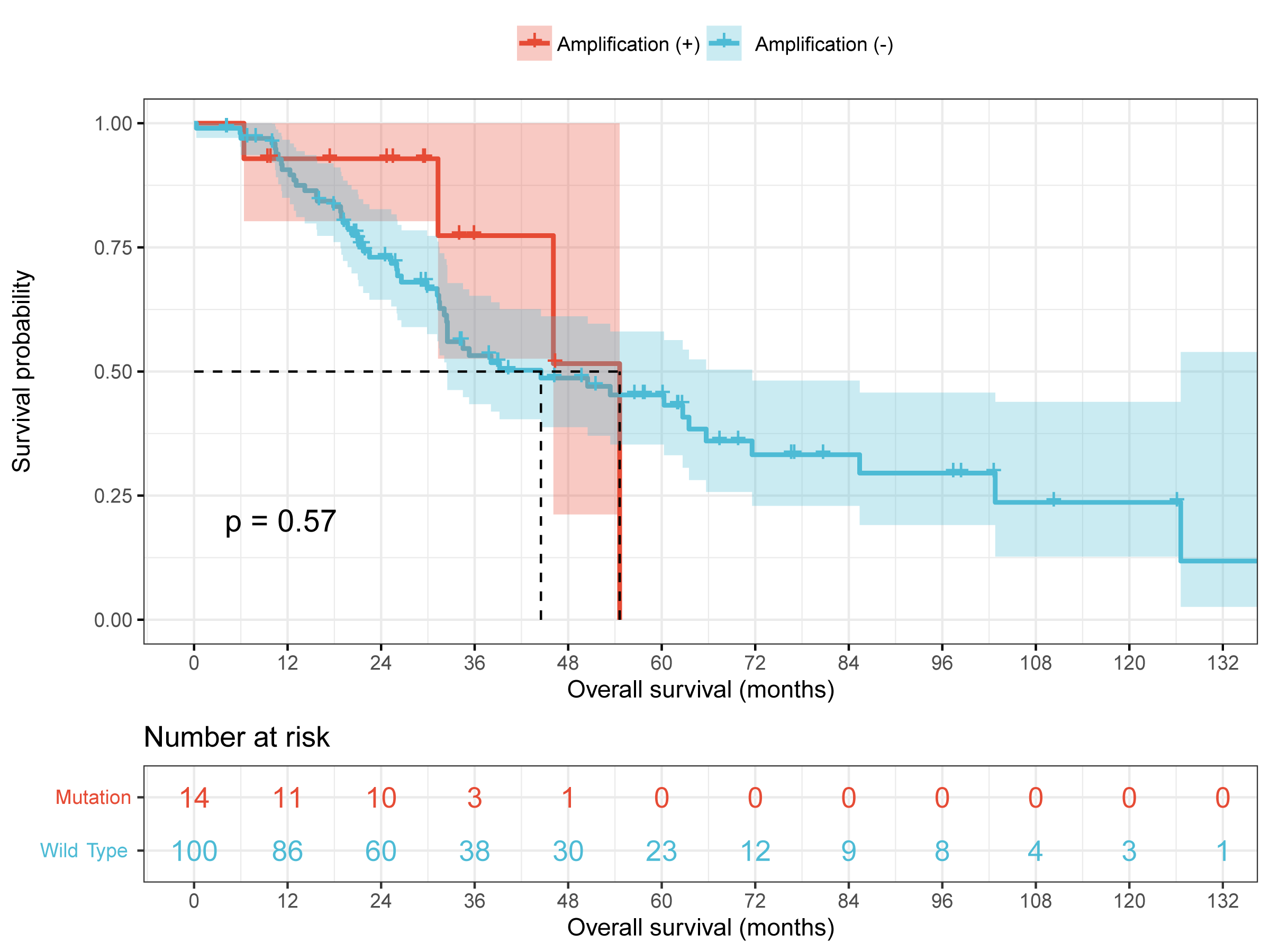

Supplement: Supplementary file 3 — Additional file 3: Supplemental Figure 2. Overall survival plot of PMS2 amplification positive and negative patients. [file 12575_2024_238_MOESM3_ESM.tif]

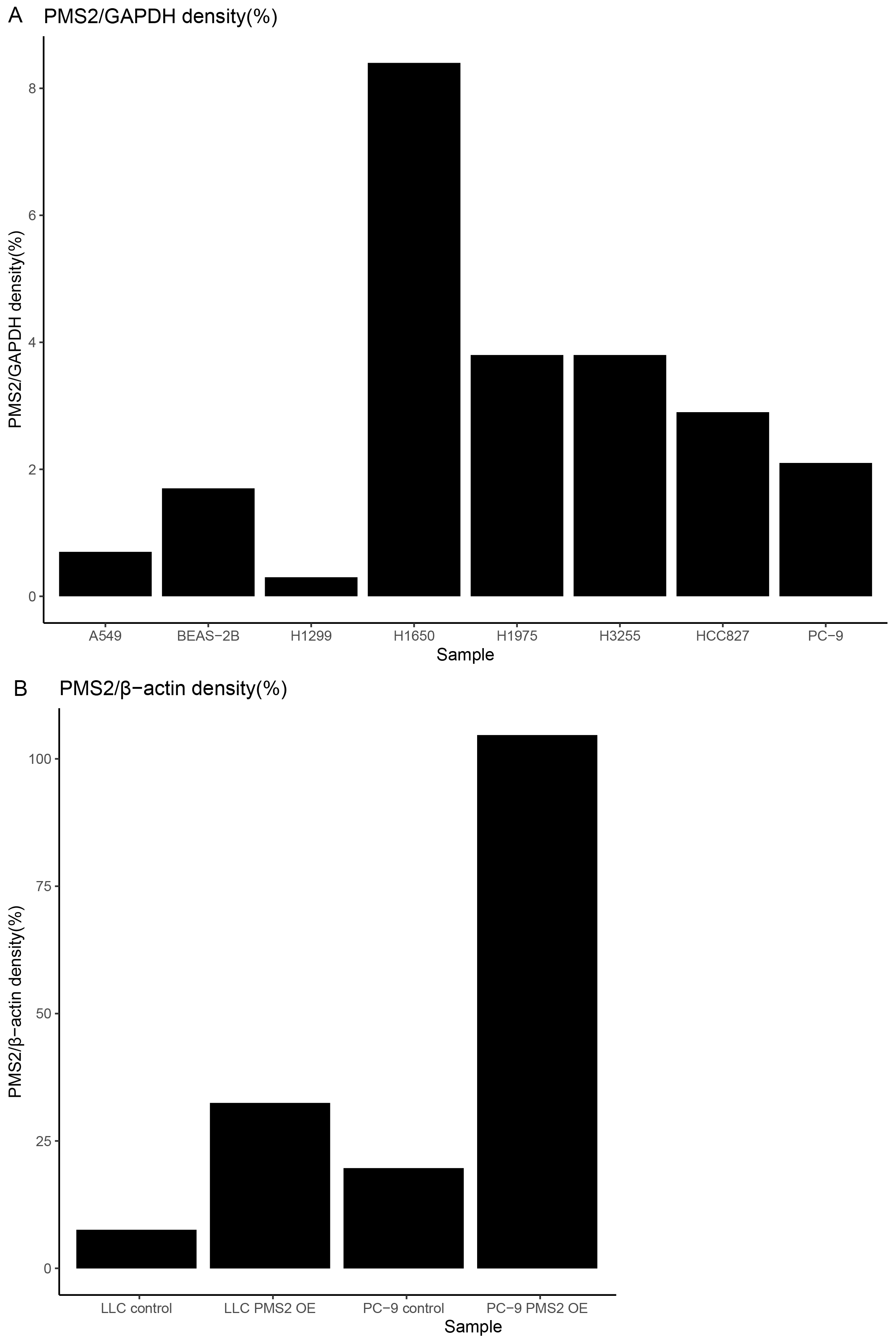

Supplement: Supplementary file 4 — Additional file 4: Supplemental Figure 3. The protein level of cell lines. (A) the ratio of PMS2 /GAPDH density in eight cell lines. (B) the ratio of PMS2 /β-actin density in transgenic lung cancer cell lines. [file 12575_2024_238_MOESM4_ESM.tif]

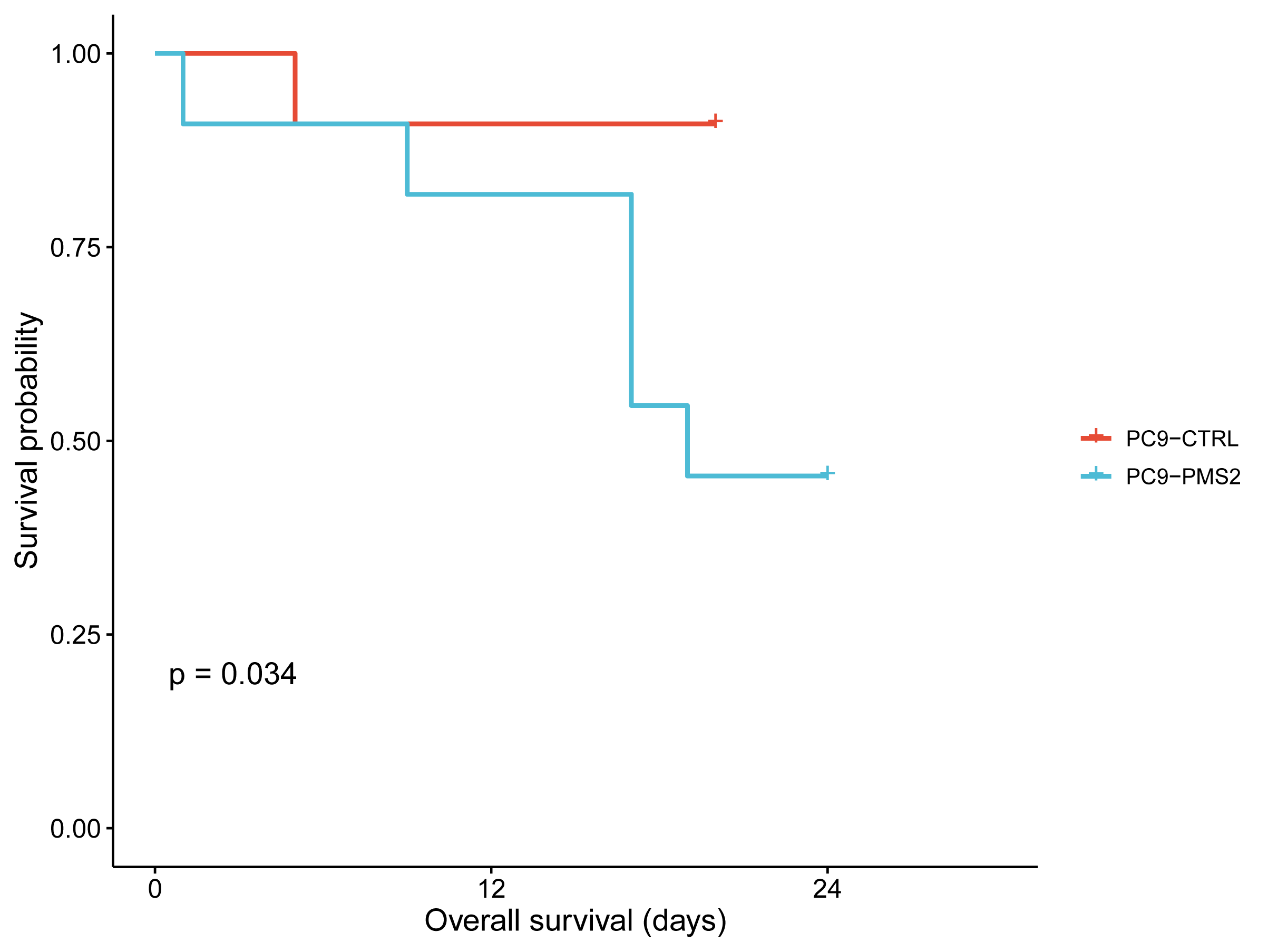

Supplement: Supplementary file 5 — Additional file 5: Supplemental Figure 4. Overall survival of mice after intracardiac injection. [file 12575_2024_238_MOESM5_ESM.tif]

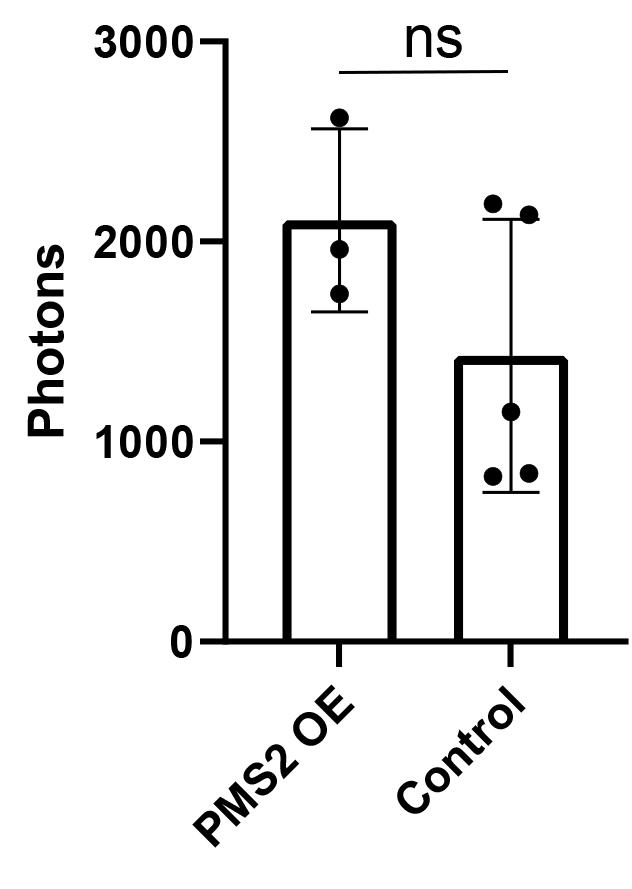

Supplement: Supplementary file 6 — Additional file 6: Supplemental Figure 5. Histogram of fluorescence signal intensity in mouse brain. [file 12575_2024_238_MOESM6_ESM.tif]

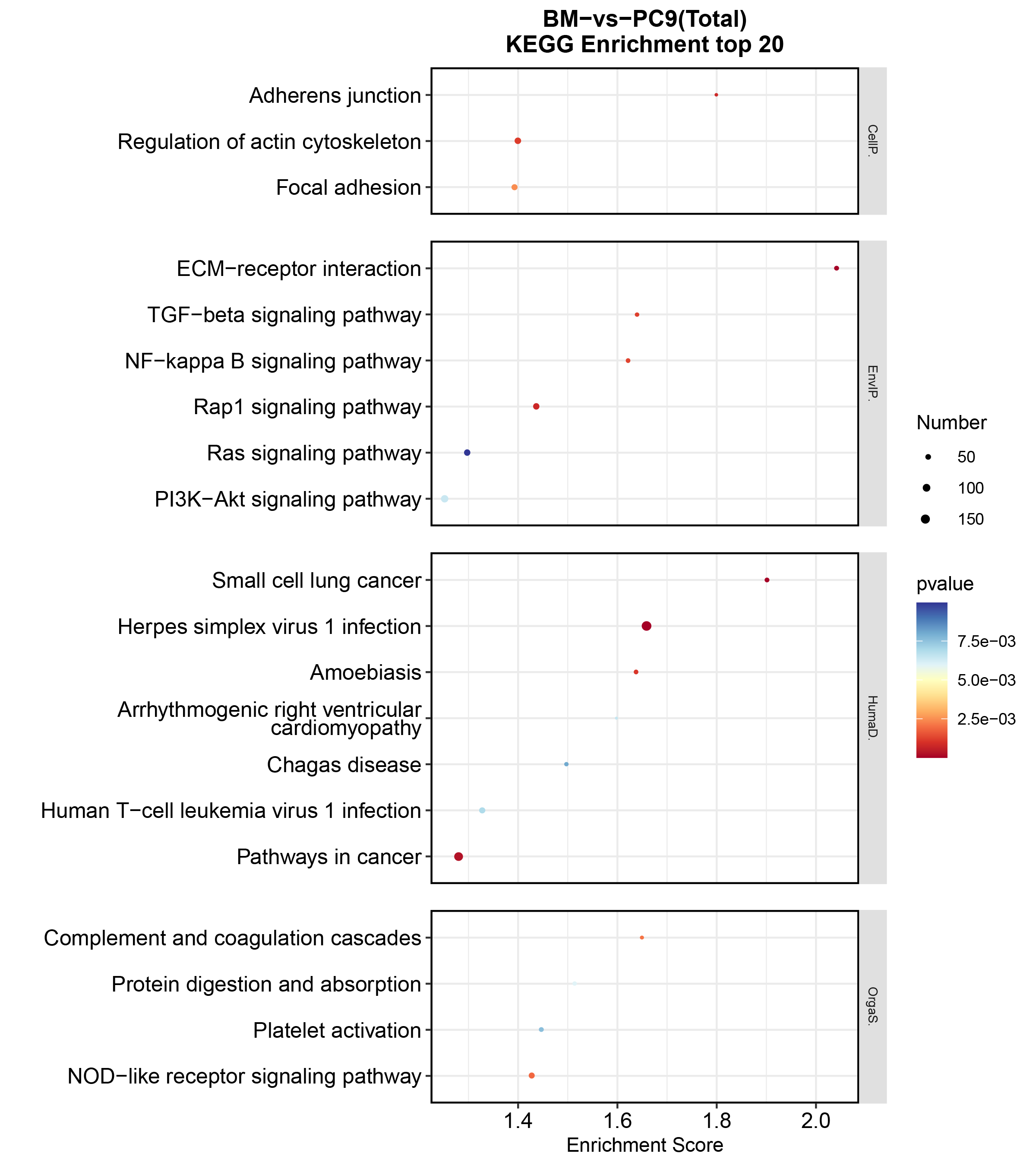

Supplement: Supplementary file 7 — Additional file 7: Supplemental Figure 6. Functional enrichment plot of PC9 cells before and after intracardiac injection. [file 12575_2024_238_MOESM7_ESM.tif]
